# How Input Parameters and Calculation Rules Influence On-Farm Antimicrobial Use Indicators in Animals

**DOI:** 10.3389/fvets.2019.00438

**Published:** 2019-12-04

**Authors:** Agnès Waret-Szkuta, Victor Coelho, Lucie Collineau, Anne Hémonic, Claire Buy, Maxime Treff, Didier Raboisson

**Affiliations:** ^1^IHAP, Université de Toulouse, INRA, ENVT, Toulouse, France; ^2^UMR EPIA INRA-VetAgroSup, Marcy-l'Étoile, France; ^3^IFIP-Institut du porc, Le Rheu, France

**Keywords:** ALEA, antimicrobials, metrics, nCD, nDD, swine, treatment incidence, weaning

## Abstract

A variety of indicators of antimicrobial use are available in veterinary medicine, their choice should depend on the study objective as none has been recognized as the most appropriate metric. Calculation of indicators of antimicrobial use is based on a number of parameters (e.g., treatment dose or weight at treatment) that can be informed using theoretical (also called “standard”) or actual (also called “used”) values. Although few studies compare the application of several indicators to the same antimicrobial data, the obtained results lead to apparent discrepancies or contradictions. This study aimed to investigate antimicrobial use at the weaning stage in French pig farms and, more specifically, the impact the sources of information regarding doses, body weight at treatment and treatment length, had on the indicators results. A cross-sectional survey was conducted, and data collected from 70 farms made it possible to calculate four indicators at the weaning stage using different input values. The indicator values did not show significant differences when calculated based on the theoretical dose and length of treatment (as recommended by the summary of product characteristics) or when calculated based on the dose used and treatment length as applied by the farmer. However, all of the indicators showed significant differences when calculated using the standard theoretical weight (15 kg) or actual weight (*P* < 0.05). It appears that if data collection plans cannot be harmonized, clarification of indicator calculations in the literature is needed to allow comparisons between studies.

## Introduction

Although numerous infectious diseases have been successfully controlled during the Twentieth century through the use of antimicrobial agents, prevention of antimicrobial resistance is a major and worldwide public health issue today (1, 2). Shared between human and animal medicine, prevention of antimicrobial resistance requires a reduction in antimicrobial use (AMU), which is the main driver for resistance (3–5). Thus, interventions that reduce AMU in food-producing animals can lead to a reduction in the presence of antimicrobial-resistant bacteria in the animal species concerned (6). A similar association is found in human populations (7, 8).

In France, a national plan named Ecoantibio 2012–2017 was enforced, aiming at a decrease in AMU of 25% over a period of 5 years. The plan encompassed 40 measures, including better monitoring of AMU, and antimicrobial resistance and harmonization of the procedure at the European and international scales (Anonymous 2011). A reduction of 37% was achieved during this period, and the new 5-year plan Ecoantibio 2 now aims at consolidating these results (9). However, measuring AMU can be quite complicated. The French National Agency for Veterinary Medicine (Agence Nationale du Médicament Vétérinaire, ANMV) monitors variations in antimicrobial sales by pharmaceutical companies yearly; however, this is not the most accurate source of data, because it considers all animal species together, and some products may be used in multiple species, including species that are not the initial target (10). A more accurate description of AMU is available from field surveys, but these are intermittent (e.g., every 3 years in pigs) (11, 12).

A variety of indicators of AMU are available in veterinary medicine, and the choice of these indicators should depend on the study objective as none has been recognized as the most appropriate metric. An indicator of AMU is defined as the amount of antimicrobials consumed normalized to the size of a population at risk of being treated in a defined period (13). Although few studies compare the application of different indicators to the same antimicrobial data, it appears that the results obtained lead to apparent discrepancies or contradictions (14–18). Not only can different methods of calculation be used for the same indicators, different data sources can also be used for each of the parameters of the corresponding formula. Thus, data can be collected at the drug producer level, the drug prescription level (veterinarian), the expenditure or delivery level or the farm level (11). The choice depends on the objective, the desired precision and the time frame as well as on the financial and human resources available to conduct the study. When calculating an indicator, information concerning the at-risk period can be variable depending on whether only the duration of the physiological status of the treated animal or its entire lifespan on the farm is considered. Likewise, weight at treatment can be estimated from the Average Daily Weight Gain (ADWG), obtained by weighting the animals or considered as equal to the European estimate of the mean weight of treated animals at a given production stage. Antimicrobial dose and treatment length can be defined by the national Summary of Product Characteristics (SPC), retrieved from veterinarian prescriptions, or reported by the farmer.

The objective of the study was (i) to describe AMU at the weaning stage in farrow-to-finish indoor pig farms in southwest France and (ii) to investigate how the choice of information sources impacts indicator results and whether the results vary depending on the indicator of interest.

## Materials and Methods

### Sampling and Data Collection

A list of 803 farrow-to-finish indoor farms present in southwest France (Nouvelle-Aquitaine and Occitanie regions) was obtained from the national database BDPORC. Two hundred and seventy-one premises declared over 40 sows; of these, 269 had either a telephone number or an email address or both. Five farms were selected randomly for the pilot study and then discarded. Of the 155 farms that could eventually be contacted within 4 phone call attempts and that complied with the inclusion criteria above, 84 farmers agreed to participate, resulting in a response rate of 54.2%. The response rates in the two regions were not significantly different (chi-2, *P*-value = 0.21).

The final sample was reduced to 70 farms due to (i) missing information (number of piglets per litter, 1 farm), (ii) missing name of the used medicine (making it impossible to find the SPC, 9 farms), and (iii) inconsistent reported values for antimicrobial dose, e.g., more than five times the SPC value (5 farms). One farm had two of the listed inconsistencies.

Data from the calendar year 2014 were collected in 2015 using a questionnaire administered in a face-to-face interview. The questions were mainly closed and were organized into 8 sections, of which three are of interest here: (i) general information on the farm, including farm size and farm management during the post-weaning stage; (ii) economic and technical results, including mean piglet weight at weaning and at the end of the weaning stage; and (iii) farm health monitoring information (number of visits per year by a veterinarian or a technician) and antimicrobial treatments administered. The details of antimicrobial treatments administered during 2014 were collected: name of the drug and percentage of active substance, route of administration, number of packages or items used, size/volume of the package or item, dose used, administration frequency, number of days the product was administered, age of the animals at the beginning of the treatment, number of animals targeted by the treatment, and type of usage. The type of usage could be either prophylactic (applied to healthy animals for the prevention of particular diseases), metaphylactic (administration of antimicrobials to animals experiencing any level of bacterial disease before overt disease occurred, with the time of intervention depending on the detection of disease outbreaks in a few animals in the group) or therapeutic (treatment only of animals showing symptoms of a disease). The dataset was therefore based on the active substances used in each treatment, on each farm and could include several active substances for a given farm.

### Indicators Calculations

**Figure 1:**
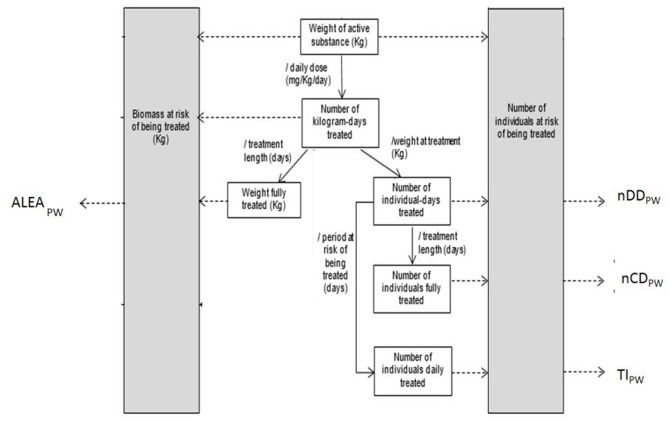
Summary of technical units of measurement indirectly accessed from number of packages or items and corresponding indicators of antimicrobial usage used [adapted from Collineau et al. (15)].

Indicators calculation formula were retrieved from Collineau (19) for nDD (number of daily doses per animal), nCD (number of entire treatment per animal), TI (number of entire treatments per day), and ALEA (Animal Level Exposure to Antibiotics). The formulas were implemented in Excel (Figure 1).

We used the following notations:

- nDD_PW_: number of daily doses per animal during the post-weaning period

$$\begin{array}{l} nDD=\;\frac{\frac{Weight\;of\;active\;substance\;\left(mg\right)}{dose\;\left(mg.kg^{-1}.day^{-1}\right)x\;weight\;at\;treatment\left(kg\right)}}{Number\;of\;individuals\;at\;risk\;of\;being\;treated} \end{array}$$

- nCD_PW_: number of entire treatments per animal during the post-weaning period

$$nCD=\;\frac{\frac{Weight\;of\;active\;substance\;(mg)}{dose\;\left(mg.kg^{-1}.day^{-1}\right)\;x\;weight\;at\;treatment\;\left(kg\right)x\;treatment\;length\;(day)}}{Number\;of\;individuals\;at\;risk\;of\;being\;treated}$$

- TI_PW_: number of entire treatments per day in the post-weaning period

$$TI=\;\frac{\frac{Weight\;of\;active\;substance\;(mg)}{dose\;\left(mg.kg^{-1}.day^{-1}\right)x\;weight\;at\;treatment\;\left(kg\right)x\;period\;at\;risk\;of\;being\;treated(day)}}{Number\;of\;individuals\;at\;risk\;of\;being\;treated}$$

- ALEA_PW_: Animal Level Exposure to Antibiotics during the post-weaning period

$$ALEA=\;\frac{\frac{Weight\;of\;active\;substance\;(mg)}{dose\left(mg.kg^{-1}.day^{-1}\right)\;x\;treatment\;length\;(day)}}{Biomass\;at\;risk\;of\;being\;treated\;(kg)}$$

Indicators were calculated for each of the 70 farms, for the post-weaning period only. The period during which the animals were at risk of being treated was considered to be equal to the duration of the post-weaning period, and the number of individuals at risk of being treated was considered to be equal to the number of post-weaning piglets produced per year (here 2014). The biomass at risk of being treated was calculated as the number of post-weaning piglets produced per year multiplied by the weight of the piglets at the end of the weaning stage. The weight of piglets at treatment used in the calculation was either obtained from the questionnaires (noted as BW, body weight) or the standard theoretical weight was used (noted as 15 kg, mean European value of 15 kg) (20). Similarly, the dose administered and the number of days of treatment were either the values reported by farmers in the questionnaires (called UDD, Used Daily Dose) or the SPC values (called ADD, Animal Daily Dose). For example, nDD_PW−ADD−BW_ was the notation used when reporting the number of daily doses per animal during post-weaning that had been calculated using actual weight at treatment, dose (ADD) and length of treatment defined by SPC. When using SPC intervals, values corresponding to therapeutic recommendations, meaning the highest dose and the shortest duration of treatment, were selected.

### Statistical Analysis

A general descriptive analysis was performed using R 3.4.1 (21). The number and proportion of records below, equal or exceeding the SPC values for either dose or length of treatment and depending on treatment usage (prophylactic, metaphylactic, therapeutic, or all together), administration route (injection, oral), medicated vs. non-medicated feed or individual vs. collective treatment and for colistin were determined. Because of the extreme differences in the doses of different antimicrobial agents (e.g.,: chlorotetracyclin 50 mg/kg and marbofloxacin 2 mg/kg), we calculated relative difference compared to the SPC value. We also considered differences between indicators calculated with (i) ADD vs. UDD and (ii) real weight at treatment (BW) vs. 15 kg (standard European theoretical weight) using non-parametric Wilcoxon paired rank tests. Alpha level for determination of significance was 0.05.

Lorentz curves were built in Microsoft Excel for each indicator calculated using (i) information retrieved from the farmer and (ii) the therapeutic recommendations to investigate whether differences in the calculation could impact farm classification in terms of the proportion of low or heavy users based on the level of AMU. Likewise, Lorentz curves were calculated using only SPC values for dose and treatment length but using (i) BW and alternatively (ii) standard European theoretical weight (15 kg). Lorentz curves represent the cumulative proportion of farms classified, ranging from the lowest antimicrobial user to the highest antimicrobial user (X axis), relative to the total AMU value in the population (of which the cumulative proportion is depicted on the Y axis). Thus, each curve is a graph showing the cumulative proportion of AMU corresponding to x% of the 58 farms having used at least one antimicrobial in 2014 and drawn from the 70 farms included in the survey. The closer a population curve is to the right corner of the graph, the more significant is the proportion of AMU that is contributed by a large sub population of farms that are low users.

## Results

### Differences Between Used and Defined Daily Doses and Treatment Lengths

The size of the 70 farms in the sample ranged from 42 to 1,083 present sows, with a mean of 172. (SD: 1.56). They represented 145 records of active substance, different records possibly corresponding to the same given active substance (Table 1). Fifty-eight (82.8%) administered at least one individual or collective antimicrobial treatment at the weaning stage in 2014. Twelve farms (17.1%) that had not used any antimicrobials in 2014 were discarded.

**Table 1:** Sample data from one of the 70 farms included in the survey: this farm used 3 treatments during post-weaning, with 3 different active substances classified in 5 records.

Line 1	Treatment 1	Active substance 1	Benzylpenicillin
Line 2		Active substance 2	Dihydrostreptomycin
Line 3	Treatment 2	Active substance 3	Amoxicillin
Line 4	Treatment 3	Active substance 1	Benzylpenicillin
Line 5		Active substance 2	Dihydrostreptomycin


Farmers used from zero to 6 different active substances during post-weaning, with a mean of 1.8 (95% CI: 1.5–2.1) and a median of 2. Nineteen different active substances were used across all farmers surveyed, corresponding to 10 different antimicrobial families with a mean of 1.7 (95% CI: 1.5–2.0), a maximum of 5 and a median of 2 antimicrobial families per farmer. Details on the active substances that were used are given in Table 2.

**Table 2:** List of active substances used by 58 of the 70 farms surveyed.

**Family**	**Active substance**	**Number of occurrences**	**Number of farms that used the active substance (% of 58)**
B Lactamin	Clavulanic acid	1	1 (1.7%)
	Amoxicillin	16	15 (25.9%)
	Ampicillin	3	3 (5.2%)
	Benzylpenicillin	4	3 (5.2%)
Aminosid	Apramycin	3	3 (5.2%)
	Dihydrostreptomycin	4	3 (5.2%)
	Neomycin	1	1 (1.7%)
	Spectinomycin	10	11 (19%)
Tetracyclin	Chlorotetracyclin	2	1 (1.7%)
	Oxytetracyclin	2	3 (5.2%)
Polymyxine	Colistin	58	50 (86.2%)
Fluoroquinolon	Enrofloxacin	5	5 (8.6%)
	Marbofloxacin	5	5 (8.6%)
Lincosamid	Lincomycin	9	12 (20.7%)
Macrolid	Tilmicosin	3	4 (6.9%)
	Tylosin	10	13 (22.4%)
Diaminopyrimidin	Trimethoprim	2	2 (3.4%)
Sulfamid	Sulfadimethoxin	3	1 (1.7%)
Pleuromutilin	Tiamulin	4	4 (6.9%)

*The number of occurrences of each active substance in the dataset is indicated along with the number and proportion of farms that reported using it in 2014*.

**Figure 2:**
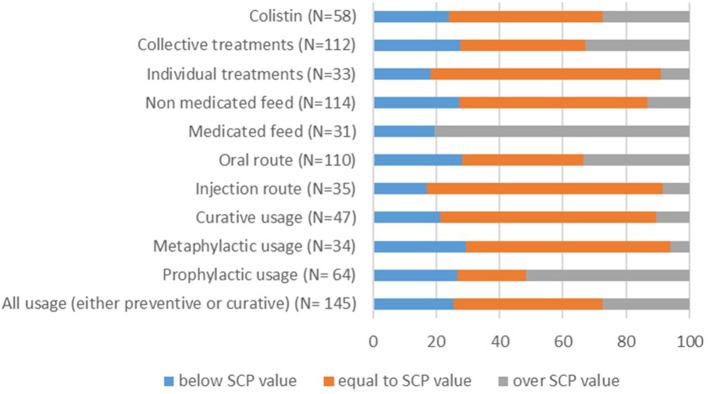
Proportion of records for which used dose (mg/kg/j) is equal to, less than or greater than the standard dose depending on treatment usage (prophylactic, metaphylactic, therapeutic, or all together), administration route (injection, oral), medicated vs. non-medicated feed, individual vs. collective treatment and for colistin. The number of total records per item (N) is indicated in brackets.

**Figure 3:**
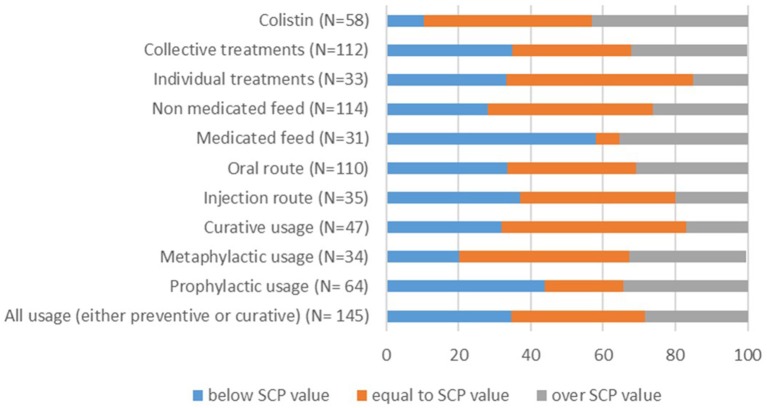
Proportion of records for which real length of treatment (days) is equal to, less than or greater than the standard length of treatment depending on treatment usage (prophylactic, metaphylactic, therapeutic, or all together), administration route (injection, oral), medicated vs. non-medicated feed, individual vs. collective treatment and for colistin. The number of total records per item (N) is indicated in brackets.

When looking at active substances that were used without distinction between usage type, 16 (11%) doses were over 150% of the SPC value. Regarding real treatment length, 49 records (33.8%) were more than 50% lower than the SPC values and 34 records (23.4%) were more than 150% higher. Sixty-four records corresponded to prophylactic usage, 34 to metaphylactic usage and 47 to therapeutic usage. For prophylactic usage, 13 records (20.3%) were higher than 150% of the SPC dose. Differences in the treatment length appeared more extreme; it was higher than 150% of the SPC value for 18 records (28.1%) and lower than 50% of the SPC value for 27 (42.2%) records. In case of therapeutic treatments, the majority of the records respected the SPC recommendations (Figures 2, 3).

Administration by the oral route was more common than injections (110 records, 75.9% vs. 35 records, 24.1%, respectively). When considering the oral route, 25 records (22.7%) were higher than 125% of the SPC value and 27 (24.5%) involved a treatment length higher than 150% of the SPC value.

There were 31 records of medicated feed vs. 114 records of non-medicated feed drugs. Length of treatment with non-medicated feed drug records was more than 150% higher than the SPC for 26 records (22.8%) and at least 50% shorter for 32 records (28.1%).

Finally, 33 records (22.8%) referred to individual piglet treatments, and 112 records (77.2%) referred to entire batch treatments. Individual treatments appeared to conform more closely to the SPC recommendations. On the other hand, doses used in group treatments were lower than 50% of the SPC value in 27 records (24.1%) and higher than 125% of the SPC value in 25 records (22.3%). In addition, 38 (33.9%) treatment lengths were lower than 50% of the SPC value, and 29 (25.9%) were higher than 150% of the SPC value.

Colistin was found to be the most frequently used active substance (58 records) and was delivered primarily through the using oral route (55 records). Colistin doses were higher than 150% of the SPC value in 4 records (6.9%). Likewise, the treatment length was lower than 50% of the SPC value in 6 records (8.6%) and higher than 150% of the SPC value in 20 records (34.5%). The variations in colistin use were similar to those observed for oral route administration, non-medicated feed, and entire batch treatment because a majority of colistin records were found for these items.

### Differences Between Used Weight and Standard Weight at Treatment

In our sample, weaning weight ranged from 5.4 to 9.0 kg, with a mean of 7.6 kg (SE: 0.8). Weight at the end of the post-weaning stage ranged from 15 to 50 kg, with a mean of 29.4 kg (SE: 6.8). Weaning stage duration ranged from 30 to 70 days, with a mean of 47.7 days. There was great variability among farms in these three parameters. The real weight at treatment in our study is equal to the mean standard European weight of 15 kg in 11.03% records (*n* = 16). Animals are lighter at the time of treatment in 78.62% of records (*n* = 114) and heavier in 10.34% of records (*n* = 15).

### Impact of Dose and Weight Choice on Indicator Value and Farm Classification

#### Impact on Indicator Value

Table 3 shows the distribution of the indicators calculated using different values for input parameters. Table 4 shows the results of the Wilcoxon rank tests.

**Table 3:** Main description of the calculated indicators of antimicrobial use (minimum, first quartile, median, third quartile, maximum, mean, standard deviation) using different information sources.

**Indicator**	**Min**	**1^st^ quart**	**Med**	**3^**rd**^ quart**	**Max**	**Mean**	**Sd**
nDD_PW−ADD−BW_	0.084	4.48	13.44	23.68	65.95	16.30	14.77
nDD_PW−UDD−BW_	0.084	2.94	12.66	22.53	89.08	18.51	21.14
nDD_PW−ADD−15kg_	0.056	2.55	7.00	15.77	49.19	11.47	12.50
nCD_PW−ADD−BW_	0.042	1.14	2.50	4.06	10.86	3.02	2.50
nCD_PW−UDD−BW_	0.033	0.90	2.76	4.37	21.09	3.45	3.91
nCD_PW−ADD−15kg_	0.022	0.73	1.60	2.91	13.20	2.28	2.66
TI_PW−ADD−BW_	0.003	0.08	0.35	0.45	1.37	0.34	0.33
TI_PW−UDD−BW_	0.003	0.06	0.23	0.48	1.81	0.40	0.47
TI_PW−ADD−15kg_	0.002	0.05	0.12	0.30	1.03	0.24	0.27
ALEA_PW−UDD−BW_	0.019	0.36	0.81	1.46	5.01	1.10	1.11
ALEA_PW−ADD−BW_	0.015	0.29	0.91	1.50	7.07	1.22	1.45

**Table 4:** Results of the non-parametric Wilcoxon paired rank test on differences between indicators calculated with (i) ADD vs. UDD and (ii) real weight at treatment (BW) vs. 15 kg (standard European theoretical weight).

	**UDD vs. ADD**	**BW vs. 15 kg**
nDD_PW_	0.94	5.87e^−9^
nCD_PW_	0.68	7.98e^−9^
TI_PW_	0.86	5.87e^−9^
ALEA_PW_	0.59	

All of the indicators showed significant differences between calculation with standard weight (15 kg) and actual weight (*P* < 0.05). There was no statistically significant difference between the values of indicators calculated using standard dose and treatment length (SCP) and those calculated using real dose and treatment length as reported by the farmer.

#### Impact on the Classification of Farms as Heavy or Low Antimicrobial Users

**Figure 4:**
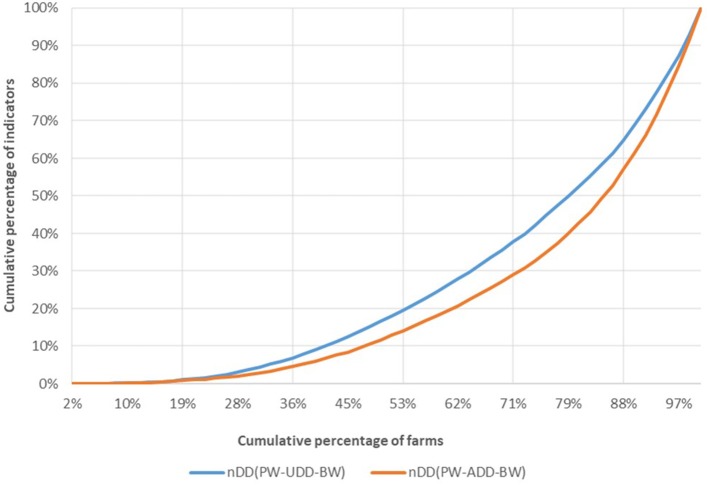
Lorentz curve: comparison between indicators calculated with ADD and UDD, indicator nDD.

Figure 4 shows the farm cumulative distribution when ADD and UDD are used in the nDD calculation.

Antimicrobial consumption is higher when farm usage (nDD_UDD_) rather than SPC recommendations (nDD_ADD_) is considered. Thus, the same amount of nDD is assumed by a smaller proportion of farms when UDD is used in the calculation (~65%) compared to the result obtained when ADD is used for the same calculus (71% of farms). This difference in indicator result concerns most of the farms for which the two curves do not overlap (~60%). The Lorentz curves for the other indicators are presented in Appendix 1 (Supplementary Data Sheet). Those curves show similar differences between the indicators calculated using UDD and those calculated using ADD, but the gaps between the curves are less important.

**Figure 5:**
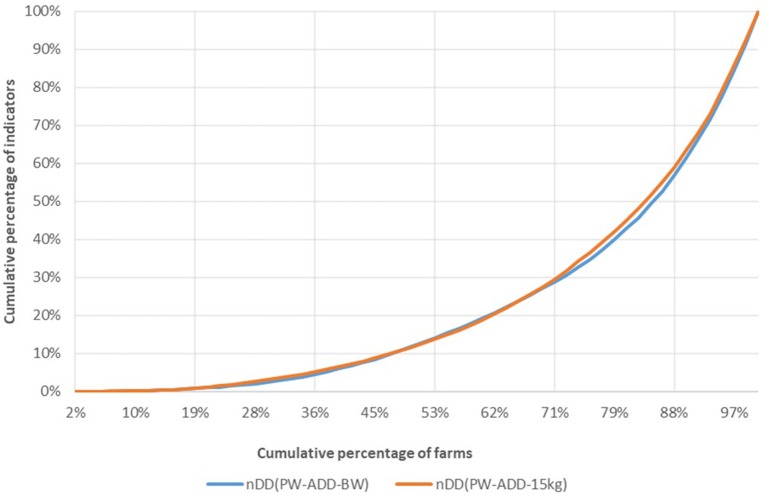
Comparison between indicators of antimicrobial usage calculated based on real weight (BW) and on European theoretical standard weight (15 kg), indicator nDD.

Figure 5 shows farm classification when real weight (BW) and standard weight (15 kg) are used in the nDD calculation.

There is no significant difference in the classification of the farms considering nDD_BW_ or nDD_15kg_. The Lorentz curves for the other indicators (Appendix 1 in Supplementary Data Sheet). show a small difference between the nCD_BW curve and the nCD_15 kg curve.

## Discussion

For indicator calculations when using SPC intervals, the highest dose and shortest treatment length were selected to maintain consistency with therapeutic recommendations but implies that present results include less compliance with SPC recommendations by farmers based upon veterinary prescription compared to field practices. Indeed, this choice influences the differences between UDD and ADD significantly and thus differences may not be regarded as a sign of “UDD was not correct.” The objective of the study was to analyze how data selection may influence indicator values and not to analyse compliance with SPC recommendations.

The size of the study sample (70 farms, 145 records) was limited by the exclusion criteria. However, the sample included farms that showed heterogeneity in size, dose, length of antimicrobial treatment applied and weight of animals. Differences in all indicators were found when calculations based on real weights and standard weights were compared, but no significant differences were found when calculations based on doses were compared. Results cannot be regarded to be representative, although specific reasons for non-representatively could not be identified. Moreover, it is not easy to obtain real and accurate information. Some farms were excluded from this study due to misreported or missing information, although all treatments are compulsorily recorded by the farmer in the farm logs as required by the EU and by national regulations (22). Therefore, a research team must choose between data that are closer to reality and data that are easier to obtain. In the short term, new tools such as GVET that offer farmers a platform for electronic recording should help (23).

The ALEA_PW_ is calculated based on a “biomass at risk of being treated” (Figure 1), and we did not analyse the impact of the choice of data for this variable. The weight chosen for the calculation of biomass can be the mean weight of piglets at weaning, the mean weight of piglets at the start of medication or the mean weight of an adult pig.

There was more non-compliance with the recommended length of treatment (72.4%) than with the recommended daily dose (53.1%). According to the Lorentz curves, the nDD is the indicator that shows the greatest difference in farm classification when used dose vs. SPC dose is considered, despite the fact that all of the indicators are influenced by the choice of used or SPC dose, as illustrated by the fact the curves do not completely overlap. We observed that the impact concerns most of the farms, but excludes those with extreme classifications (low or heavy users). The use of ADD for calculation leads to a lower result, and thereby to an underestimation of AMU.

The choice of real or European standard theoretical weight at treatment has a smaller influence on the final classification than the choice of dose and length of treatment. The nCD was the most influenced indicator, and, similar to the findings regarding dose and length of treatment, the choice regarding weight data primarily impacts the middle-user farms rather than the extreme antimicrobial users. Thus, the use of a standard weight for calculation leads to a lower result as well as to an underestimation of AMU. In France, this can be linked to the fact that most treatments are administered at the beginning of the weaning stage (management of diarrhea post-weaning) to piglets weighing less than the ESVAC reference.

Moreover, all countries do not use the same SPC values, these values can be very different, and may question the definition of good therapeutic practices (24). Thus, it could be recommended that real dose, treatment length and real weight be used whenever possible to analyse antimicrobial consumption. Using real values would allow a better description of actual exposure to antimicrobials, although today one would prefer using standard references when aiming at comparisons.

Data from 2014 were collected in southwest France in 2015. Prophylactic usage of antimicrobials, which are still used at high levels in many countries to sustain animal health and welfare was recently banned in feed for farm animals in the EU (25), and these were the treatments for which most discrepancies between ADD and UDD were found in our study.

Colistin use appeared high in this study because the data were collected in 2014, since then, colistin use has drastically decreased in accordance with EMA recommendations (26).

The oral route was more commonly practiced than injection which can be easily explained by the ease of application and the challenge of identifying sick animals in a population, considerations that are involved when managing the effective use of drugs (27). Many pathogens also affect whole groups of pigs, even subclinically, and in such cases the whole group needs to receive antimicrobial medication (28). However, there are issues with administering group medication through the water supply or through feed, mainly with respect to (i) the inter- and intra-individual variability in drinking and feeding behavior and the resulting variability in actual intake of dose; (ii) the risk of AMR damaging the animals' microbiota. In two herds of our sample, the farmers managed to practice injection on whole batches which seems to be a valuable evolution in terms of tackling AMR, although it does not appear as an ideal solution on its own. The use of this method is supported in our study by the fact that injections were practiced in a manner that more closely followed SPC recommendations. Precision livestock farming would be expected to offer opportunities to limit injection-related risks, pain to the animals and costs to the farmer by allowing the early detection of diseased animals.

Our survey did not include questions related to antimicrobial use in sows, piglets during lactation, or pigs during the fattening period. It might have been interesting to investigate whether a low user at the weaning stage was a high user during fattening, for example, which would also have enabled the AMU values found in this study to be compared with those reported in other studies. However, we aimed to maximize the accuracy of data collection by focusing on the weaning stage, which has been identified as the critical period for AMU in pigs. This should enable as a next step the investigation and ordering of risk factors as a basis for proposing practical measures to be implemented in the field to continue decreasing AMU.

## Data Availability Statement

The datasets generated for this study are available on request to the corresponding author.

## Ethics Statement

The studies involving human participants were reviewed and approved by Comité d'Ethique de l'Ecole Nationale Vétérinaire de Toulouse. The patients/participants provided their written informed consent to participate in this study in accordance with the Declaration of Helsinki.

## Author Contributions

AW-S designed the study and performed the analysis along with VC and DR. VC drafted the first version of the manuscript. CB participated in designing of the questionnaire and analyzed the pilot study. VC, CB, and MT collected the data in the field. AW-S, VC, LC, AH, CB, MT, and DR contributed significantly to the discussion, read, and approved the manuscript.

### Conflict of Interest

The authors declare that the research was conducted in the absence of any commercial or financial relationships that could be construed as a potential conflict of interest.

## Abbreviations

ADG: average daily weight gain
AMU: antimicrobial use
BW: body weight
SPC: summary of product characteristics
nDD: number of daily doses per animal
nCD: number of entire treatments per animal
TI: number of entire treatments per day
ALEA: animal level exposure to antibiotics
UDD: used daily dose: dose and treatment length declared by the farmer
ADD: animal daily dose: dose and treatment length defined by SCP.
