# Supplementary material for: How Input Parameters and Calculation Rules Influence On-Farm Antimicrobial Use Indicators in Animals
Source: Front Vet Sci. 2019 Dec 4;6:438. doi: 10.3389/fvets.2019.00438 (PMC6904276; doi:10.3389/fvets.2019.00438)
Supplement: Supplementary file 1 [file Data_Sheet_1.docx]

Annex 1

Figure comparing dose and number of days of treatment for nCD indicator using real weight (BW)

Figure comparing dose and number of days of treatment for TI indicator using real weight (BW)

Figure comparing dose and number of days of treatment for ALEA_PW_ indicator using real weight (BW)

Figure comparing use of European theoretical weight (15kg) and real weight (BW) for nCD calculation using standard dose and treatment length

Figure comparing use of European theoretical weight (15kg) and real weight (BW) for TI calculation using standard dose and treatment length
